# 25-Hydroxyvitamin D Concentration and Leukocyte Telomere Length in Young Adults: Findings From the Northern Finland Birth Cohort 1966

**DOI:** 10.1093/aje/kwv203

**Published:** 2016-01-23

**Authors:** Dylan M. Williams, Saranya Palaniswamy, Sylvain Sebert, Jessica L. Buxton, Alexandra I. F. Blakemore, Elina Hyppönen, Marjo-Riitta Jarvelin

**Keywords:** biological aging, Northern Finland Birth Cohorts, telomere length, vitamin D

## Abstract

Higher vitamin D status, lower adiposity, and longer telomere length are each reportedly associated with lower risk of several chronic diseases and all-cause mortality. However, direct relationships between vitamin D status (measured by circulating 25-hydroxyvitamin D (25(OH)D) concentration), adiposity, and telomere length are not well established. We conducted a cross-sectional analysis of associations of 25(OH)D and body mass index (BMI; weight (kg)/height (m)^2^) with mean relative leukocyte telomere length (LTL) using data gathered on 5,096 participants from Northern Finland Birth Cohort 1966 at age 31 years (1997). 25(OH)D was not associated with LTL in either basic or confounder/mediator-adjusted models. BMI was inversely associated with LTL after adjustment for potential confounding by age, sex, socioeconomic position, physical activity, diet, smoking, alcohol intake, and use of oral contraceptives (per 1-unit increase in BMI, mean difference in LTL = −0.4%, 95% confidence interval: −0.6, −0.2). The BMI-LTL association was also independent of 25(OH)D and was attenuated slightly, but remained, after adjustment for C-reactive protein, a marker of low-grade inflammation (mean difference in LTL = −0.3%, 95% confidence interval −0.6, −0.1). These findings suggest that vitamin D status is unlikely to be an important determinant of LTL, at least by young adulthood. Inflammation may partly mediate associations of adiposity with LTL.

In addition to a well-established role in bone metabolism, the active form of vitamin D (calcitriol) is purported to have a range of biological effects in many types of nonskeletal tissue and cells (1). Epidemiologic studies have found associations of higher vitamin D status, as measured by circulating 25-hydroxyvitamin D (25(OH)D) concentration, with a lower risk of all-cause mortality and several nonskeletal diseases, including cardiovascular disease and cancer (2–4), albeit with many equivocal and uncertain findings (5). Evidence from randomized controlled trials is currently insufficient for drawing conclusions on causality for most disease outcomes (6), but trial evidence does suggest that small-dose vitamin D supplementation may lead to lower all-cause mortality risk (6). These findings are supported by evidence from a Mendelian randomization analysis of potential causal effects of 25(OH)D on mortality (7). Thus, if vitamin D status is causally related to mortality risk, it may be exerting effects via 1 or more biological pathways, including the maintenance of telomere length.

Telomeres are nucleoprotein “caps” formed of repetitive DNA sequences and specialized proteins at the ends of chromosomes (8). They protect chromosomes from deterioration and fusion during mitosis (9). Telomeres are progressively eroded with successive rounds of cell division and by attrition, attributed to processes such as oxidative stress (10). There is variation in telomere length between persons of equal chronological age, due to differences in both genetic factors and environmental determinants (including adiposity) (11–14). Shorter age-adjusted leukocyte telomere length (LTL), a standard measure of individuals' telomeres, is associated with a range of chronic conditions, including cardiovascular disease (15) and type 2 diabetes (16). Shorter telomeres are also associated with an increased risk of several cancers, including bladder cancer and gastric cancer (17), although longer LTL is associated with increased risk of melanoma and some other cancers (18). Despite the complex relationship between telomere length and cancer risk, LTL is positively associated with life span and inversely associated with several age-related disorders, largely reflecting the associations observed for 25(OH)D (19).

Vitamin D status could plausibly affect the maintenance of telomere length directly or via effects on influencing mechanisms, such as inflammation and/or the rate of cell proliferation (20–23). If higher vitamin D status rate-limits telomere attrition, 25(OH)D concentration could represent a modifiable target for improving maintenance of telomere length over the life course. Two large cross-sectional studies have examined 25(OH)D concentration in relation to LTL in samples of healthy females (both pre- and postmenopausal) (24, 25). Both found that higher 25(OH)D was associated with longer LTL. However, to our knowledge, no study has yet presented findings from a large population sample of both males and females. It is also unclear whether such an association is present in samples of younger adults, since the previous studies had participants with mean ages of approximately 49 and 59 years.

In this study, we used data collected at 31 years of age on 5,096 male and female participants from Northern Finland Birth Cohort 1966 (NFBC1966), with the following aims: 1) to search for cross-sectional associations of 25(OH)D with LTL; 2) to assess whether the inverse cross-sectional association of body mass index (BMI) with LTL previously reported in this cohort was independent of individual 25(OH)D concentrations (26); and 3) to test whether any observed associations of 25(OH)D and BMI with LTL were independent of concentrations of C-reactive protein (CRP), a marker of low-grade inflammation (hypotheses are illustrated in Figure 1).

**Figure 1.**
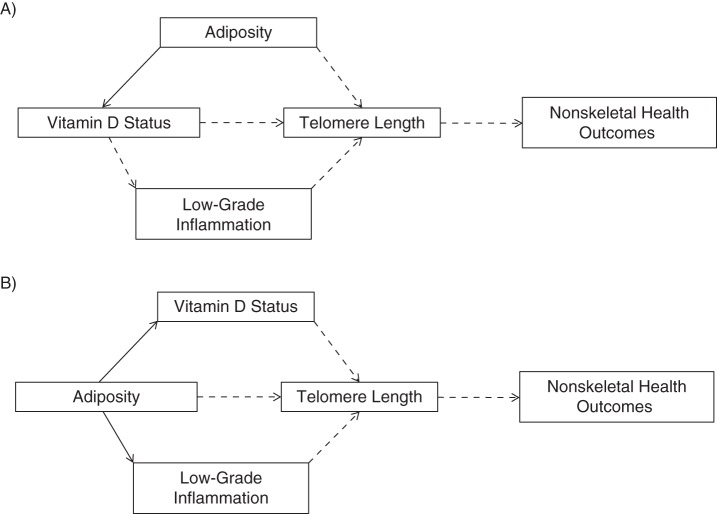
Directed acyclic graphs illustrating the 2 main hypotheses being tested in 5,096 participants from Northern Finland Birth Cohort 1966, 1966–1997. The hypothesized directions of causality are represented by dashed arrows, and established causal links are shown by solid arrows. A) A scenario in which higher 25-hydroxyvitamin D (25(OH)D) status is regarded as a potential determinant of longer leukocyte telomere length (LTL), which in turn may affect risk of health outcomes. Adiposity would be regarded as a confounder of an association between 25(OH)D and LTL, given that adiposity is a known determinant of 25(OH)D concentrations and a potential determinant of LTL (independent of effects on 25(OH)D). Inflammation may mediate an association between 25(OH)D and LTL, based on evidence that vitamin D may have antiinflammatory effects and that low-grade inflammation may shorten LTL over time. (Other mechanisms by which vitamin D could affect health outcomes, besides potential effects on LTL, were not investigated here, nor were we investigating associations of LTL with health outcomes.) B) A scenario in which higher adiposity is regarded as a determinant of shorter LTL, in turn leading to effects on health outcomes. In this model, an association of adiposity with LTL may be mediated by effects of adiposity on 25(OH)D concentrations and/or inflammation, or via other mechanisms (direct arrow from adiposity to LTL).

## METHODS

### Participants

**Figure 2.**
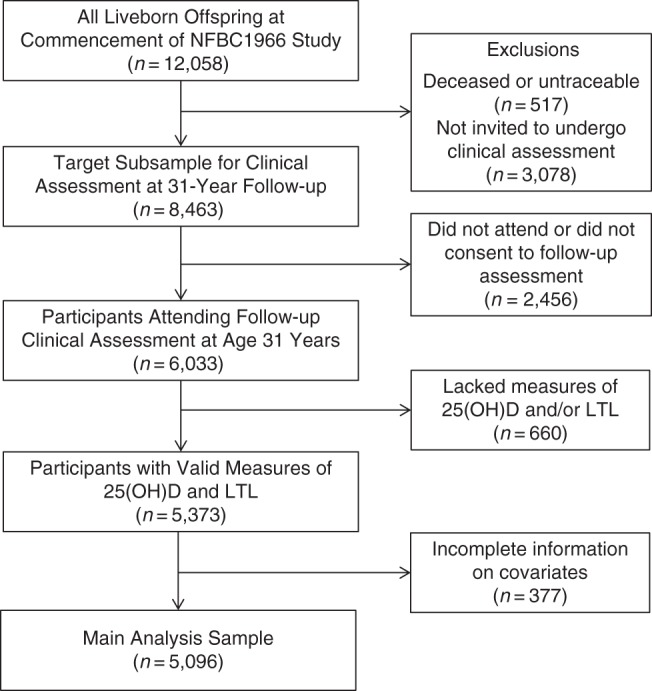
Derivation of the analysis sample from Northern Finland Birth Cohort 1966 (NFBC1966), 1966–1997. LTL, leukocyte telomere length; 25(OH)D, 25-hydroxyvitamin D.

The NFBC1966 is a prospective birth cohort study that aimed to recruit all pregnant women living in the provinces of Oulu and Lapland in northern Finland with expected delivery dates in 1966 (27). A total of 12,058 liveborn offspring, all of white European ethnic origin, were enrolled in the cohort. Detailed information was collected on them and their parents, starting prenatally and with further follow-up thereafter. In 1997, all living offspring (at age 31 years) with known addresses were contacted and sent a postal questionnaire. A subset of 8,463 offspring residing in the area of Oulu, Lapland, or Helsinki were also invited to undergo a clinical assessment, which included blood sampling. Of these participants, 6,033 attended the assessment. Informed written consent for the use of the data was obtained, and approval was granted by the Ethics Committee of the Northern Ostrobothnia Hospital District in Oulu, Finland, in accordance with the Declaration of Helsinki. For this study, a total of 5,096 participants had valid measures of 25(OH)D, LTL, and all covariates of interest; these persons formed our study sample (see Figure 2).

### Measures

#### 25(OH)D concentration

Blood samples drawn at the 31-year follow-up assessment in 1997 were collected in the morning (between 8:00 am and 11:00 am) following an overnight fast by participants. Samples were stored at −70°C until analyzed. In 2008, frozen samples were thawed, and concentrations of 25-hydroxyvitamin D_2_ (25(OH)D_2_) and 25-hydroxyvitamin D_3_ (25(OH)D_3_) were measured in 4 batches using high-performance liquid chromatography–tandem mass spectrometry. Total 25(OH)D concentration was derived from the sum of 25(OH)D_2_ and 25(OH)D_3_ concentrations. Further assay details are provided in the Web Appendix, available at http://aje.oxfordjournals.org/.

#### Leukocyte telomere length

Mean relative LTL was measured in genomic DNA samples prepared from peripheral blood leukocytes in 31-year assessment samples, using a multiplex quantitative real-time polymerase chain reaction (qPCR) method (28), with modifications as described previously (29). Briefly, the multiplex qPCR method is based on a measure of the amplification of the telomeric DNA sequence (T) relative to that of a single-copy gene (S) in each test sample, and normalized using a common reference DNA sample. This provides “T/S ratios” for each DNA sample, which are used as mean relative LTL values for participants. The overall mean coefficient of variation for T/S values of duplicate test samples on the same plate was 5%, and the mean interrun coefficient for selected samples was 6.2%.

#### Covariates

The following variables were regarded as potential confounders of associations of 25(OH)D and BMI with LTL: age, sex, BMI (in 25(OH)D-LTL models), socioeconomic position (SEP), diet quality, smoking, alcohol consumption, physical activity, and oral contraceptive use (women only) (12–14, 30–37). CRP was investigated as a potential mediator of associations.

Height and weight were measured at the assessment or derived from self-reported questionnaire responses (0.6% of the sample). BMI was calculated as weight (kg) divided by height squared (m^2^).

Information on SEP, diet, smoking, alcohol intake, physical activity, and type of contraceptive used (women only) was recorded from responses to the postal questionnaire sent as part of the 31-year follow-up. SEP was defined from responses to questions on occupation and employment status, with the following categories used: unskilled worker, skilled worker, professional, farmer, or other (retired, student, or long-term unemployed). A 6-category diet quality variable was calculated (ranging from healthiest quality (0) to unhealthiest quality (5)) based on reported frequency of intake of the following food groups in the previous 6 months: grains, dairy foods, vegetables, fruits, meat, fish, and eggs, and several miscellaneous food types (38). Alcohol consumption was calculated as a continuous variable (ethanol intake in g/day) from detailed questions on type, strength, and frequency of alcoholic drinks consumed. Physical activity level (metabolic equivalent of task (MET)-hours/week) was calculated from in-depth questions on the frequency of various forms of light and strenuous activities undertaken (39). Smoking status was derived from several questions: ever/never smoking, current number of cigarettes per day for current smokers (continuous scale), and current use or nonuse of alternative tobacco products (cigars, pipes, snuff, and chewing tobacco). Three variables for these responses were entered as covariates into models to capture a higher degree of information on smoking habits. A binary variable was derived for whether or not female participants used oral contraceptives, which interfere with vitamin D metabolism and may also alter inflammatory processes (and hence could affect LTL) (32, 40).

Serum high-sensitivity CRP concentrations were determined by means of an immunoenzymometric assay (Medix Biochemica, Espoo, Finland). The intra- and interassay coefficients of variation were 4.2% and 5.2%, respectively.

### Statistics

To account for seasonal variability, we adjusted 25(OH)D levels for season of sampling by modeling values against their dates of sampling with trigonometric sine and cosine functions (see Web Appendix for more information). We used multivariable linear regression models to examine associations of 25(OH)D and BMI with LTL and to adjust for potential confounding and mediating factors. Natural logarithmic transformation was applied to telomere T/S ratio to obtain a normal distribution of this variable as the outcome variable in regression models. A random-effects term (calculated by maximum likelihood estimation) was included in all models to account for batch effects from LTL assay plates. Regression coefficients and 95% confidence intervals were expressed in terms of relative percent change in LTL per 1-nmol/L increase in 25(OH)D concentration or per unit increase in BMI, by reformatting ratios of geometric means and 95% confidence intervals into percentages.

We fitted several multivariable models for examining each exposure-outcome association. For examination of 25(OH)D-LTL associations, model 1 included adjustment for exact age at assessment (in days), sex, and 25(OH)D sampling batch. In model 2, we included additional adjustments for potential confounders: BMI, SEP, diet quality, physical activity, smoking status, alcohol consumption, and use of oral contraceptives. In model 3, we also adjusted for CRP. To examine BMI-LTL associations, model 1 included adjustment for age and sex only. Model 2 also included adjustment for SEP, physical activity, diet, smoking status, alcohol intake, and use of oral contraceptives. Adjustments for 25(OH)D and 25(OH)D sampling batch were additionally added in model 3. Model 4 included adjustment for CRP in addition to the adjustments included in model 3.

Given the possibility of sex-specific associations of 25(OH)D and BMI with LTL and associations that the use of oral contraceptives could have with both 25(OH)D and LTL (perhaps via modification of inflammation exposure, for example) within females, we tested for interactions with sex in 25(OH)D-LTL analyses and BMI-LTL analyses, as well as interactions with oral contraceptive use (women only).

We tested for possible nonlinearity of associations between exposures and outcomes by examining fractional polynomial statistics and graphical plots. To evaluate whether there were more pronounced differences in LTL among persons with large differences in 25(OH)D concentrations, we fitted multivariable regression models comparing mean LTL in persons with 25(OH)D levels under 50 nmol/L and between 50 nmol/L and 75 nmol/L with mean LTL in those with 25(OH)D levels over 75 nmol/L. We also repeated 25(OH)D analyses without adjusting 25(OH)D for season of sampling, in order to obtain results more comparable to those of a previous cross-sectional study of relevance to our hypotheses that did not apply seasonal adjustment (25). In case there were inaccuracies in the qPCR measurement of extreme LTL values, we repeated the main analyses with LTL-value outliers excluded (≥3 standard deviations from the mean of the log*_e_*-transformed T/S ratio distribution).

## RESULTS

Table 1 shows characteristics of NFBC1966 participants in the analysis sample, along with corresponding information on those excluded because of missing data. In general these groups were comparable; there was evidence that persons in the analysis sample had higher alcohol consumption and lower LTL than those excluded, but numerical differences for these characteristics were small, and the groups did not differ on average in any other respect. Mean 25(OH)D concentration in the analysis sample (50.6 nmol/L) was similar to previously reported mean concentrations in a comparable study sample from southern Finland (47 nmol/L and 45 nmol/L in women and men, respectively; probably lower because 25(OH)D was measured at the seasonal nadir of 25(OH)D concentration in late winter/early spring) (41).

**Table 1.** Characteristics of Participants Included in Analyses and of Those Excluded Because of Missing Data on 1 or More Variables, Northern Finland Birth Cohort 1966, 1966–1997

Characteristic	Included (*n* = 5,096)		Excluded (*n* ≤ 937)		*P* Value^a^
	Median (IQR)	%	Median (IQR)	%	
Total 25(OH)D level, nmol/L^b^	50.6 (14.9)		49.8 (15.3)		0.21
Age, years^b^	31.1 (0.35)		31.1 (0.34)		0.12
Female sex		51.8		53.7	0.27
Body mass index^c^	24.0 (21.9–25.6)		24.1 (21.9–26.9)		0.32
Socioeconomic position					0.58
Farmer		3.6		3.5	
Professional		23.8		23.3	
Skilled worker		31		29.8	
Unskilled worker		25.6		25.3	
Other		15.8		18.1	
Physical activity, MET-hours/week	11.3 (3.8–20.3)		10.5 (3.8–22.5)		0.72
Diet quality^d^					0.41
0		7.5		7.1	
1		24.3		25.8	
2		30.4		27.9	
3		26.6		25.9	
4 or 5		11.3		13.3	
Ever smoking, yes/no		63.2		64.1	0.64
Current smoking (*n* = 1,838), cigarettes/day	10 (5–20)		12 (7–20)		0.28
Use of alternative types of tobacco^e^		3.4		4.1	0.31
Alcohol consumption, g/day	4.2 (1.1–11)		3.6 (0.9–8.7)		0.002
Use of oral contraceptives (women only)		23.6		21.3	0.26
C-reactive protein level, mg/L	0.75 (0.36–1.87)		0.78 (0.38–2.14)		0.17
Leukocyte telomere length, T/S ratio	1.13 (0.91–1.41)		1.16 (0.95–1.46)		0.03

Abbreviations: IQR, interquartile range; MET, metabolic equivalent of task; 25(OH)D, 25-hydroxyvitamin D.

^a^ *P* for difference between excluded and included participants, based on a 2-tailed *t* test, Kruskall-Wallis test, or χ^2^ test.

^b^ Values are presented as mean (standard deviation) because of the variable's approximately normal distribution.

^c^ Weight (kg)/height (m)^2^.

^d^ Lower values indicate a healthier diet.

^e^ Cigars, pipes, snuff, or chewing tobacco.

Web Table 1 shows the characteristics of NFBC1966 participants across the 25(OH)D distribution. There were increasing trends in physical activity, diet quality, alternative tobacco use, and use of oral contraceptives by women across increasing fifths of 25(OH)D concentration. There was also an approximately linear decreasing trend in BMI across increasing fifths, and SEP category patterning varied across the 25(OH)D distribution. No trends were observed for age, sex, cigarette smoking variables, CRP, or LTL.

Table 2 shows results from multivariable regression models of associations of 25(OH)D with LTL. There was no evidence for an association in model 1, where results were adjusted only for age and sex. Results were essentially unchanged after further adjustments for potential confounders and mediators in models 2 and 3.

**Table 2.** Associations of 25-Hydroxyvitamin D With Leukocyte Telomere Length in Adults Aged 31 Years (*n* = 5,096) From Northern Finland Birth Cohort 1966, 1966–1997

	Mean Difference in LTL, %^a^	95% CI	*P* Value
Model 1^b^	−0.01	−0.2, 0.2	0.94
Model 2^c^	−0.01	−0.2, 0.2	0.91
Model 3^d^	−4 × 10^−5^	−0.2, 0.2	0.97

Abbreviations: CI, confidence interval; LTL, leukocyte telomere length; 25(OH)D, 25-hydroxyvitamin D.

^a^ Relative percent change in LTL (T/S ratio) per 1-nmol/L increase in 25(OH)D.

^b^ Results were adjusted for age, sex, and 25(OH)D batch.

^c^ Results were adjusted for the factors in model 1 plus body mass index, socioeconomic position, physical activity, diet, smoking status, alcohol intake, and use of oral contraceptives (women only).

^d^ Results were adjusted for the factors in model 2 plus C-reactive protein.

Multivariable associations of BMI with LTL are shown in Table 3. BMI was inversely associated with LTL in model 1, adjusted for age and sex. The strength of association was almost identical with further adjustment for SEP, diet, physical activity, smoking status, and alcohol consumption (model 2) and additional adjustment for 25(OH)D (model 3). The association was attenuated slightly with additional adjustment for CRP, but it remained strong (model 4).

**Table 3.** Associations of Body Mass Index^a^ With Leukocyte Telomere Length in Adults Aged 31 Years (*n* = 5,096) From Northern Finland Birth Cohort 1966, 1966–1997

	Mean Difference in LTL, %^b^	95% CI	*P* Value
Model 1^c^	−0.4	−0.6, −0.2	0.001
Model 2^d^	−0.4	−0.6, −0.2	0.001
Model 3^e^	−0.4	−0.6, −0.2	<0.001
Model 4^f^	−0.3	−0.6, −0.1	0.01

Abbreviations: CI, confidence interval; LTL, leukocyte telomere length; 25(OH)D, 25-hydroxyvitamin D.

^a^ Weight (kg)/height (m)^2^.

^b^ Relative percent change in LTL (T/S ratio) per unit increase in body mass index.

^c^ Results were adjusted for age and sex.

^d^ Results were adjusted for the factors in model 1 plus socioeconomic position, physical activity, diet, smoking status, and alcohol intake.

^e^ Results were adjusted for the factors in model 2 plus 25(OH)D and 25(OH)D batch.

^f^ Results were adjusted for the factors in model 3 plus C-reactive protein.

There was no evidence for an interaction between 25(OH)D and BMI in associations with LTL (*P* = 0.55). There was also no evidence for interactions in the association of either exposure with LTL by sex or oral contraceptive use in the subsample of females only (all *P*'s ≥ 0.39). There was no evidence that the associations examined deviated from linearity (all fractional polynomial *P*'s ≥ 0.36).

Web Table 2 shows results from models comparing mean LTLs by category of 25(OH)D concentration. Consistent with findings from linear models, there was no evidence of differences in LTL in lower categories of 25(OH)D (<50 nmol/L or 50–75 nmol/L) compared with the highest category (>75 nmol/L).

Models using 25(OH)D without adjustment for season of sampling did not differ notably from the main results shown in Table 2 (data available upon request). Exclusion of extreme values from the LTL distribution (≥3 standard deviations) produced no differences in the results of the main models (data available upon request).

## DISCUSSION

The findings of this study do not support the hypothesis that higher circulating 25(OH)D concentrations are associated with longer LTL in young adults. Moreover, we have shown that the previously reported inverse association of BMI and LTL in these data is independent of individuals' 25(OH)D status and partly independent of low-grade chronic inflammation. Our finding that the BMI-LTL association was attenuated slightly with adjustment for CRP supports the possibility that 1 or more aspects of inflammation partly mediate any causal effects of adiposity on the maintenance of telomere length, which is consistent with past findings (42).

The lack of an association of 25(OH)D with LTL in this study contrasts with findings from 2 previous cross-sectional studies. In a study of 2,160 female twins in the United Kingdom (mean age = 49.4 (standard deviation, 12.9) years), 25(OH)D was positively associated with LTL after adjustment for age, season of 25(OH)D sampling, menopausal status, use of hormone replacement therapy, physical activity, and the twins' family structure (24). Several other potential confounders (BMI, smoking, and circulating insulin and CRP concentrations) were not included as adjustments following a Bayesian model selection. In a study of 1,337 female registered nurses in the United States (mean age = approximately 59 years), Liu et al. (25) also reported a positive association of 25(OH)D with LTL after adjustment for age, smoking status, BMI, and physical activity. It is not obvious why our findings differ from these. Unlike those analyses, this study's sample included men and women, but there were no differences in associations of 25(OH)D with LTL by sex (i.e., null findings for both), and the large size of our sample meant that we would have had adequate power to detect a magnitude of association similar to that reported previously from data on women alone. Many of the characteristics were similar between samples, such as participants being exclusively or predominantly of white European ethnicity. It is possible that we did not observe an association of 25(OH)D with LTL in this study because its sample was of a notably younger average age (mean age = 31 years). Associations of 25(OH)D with cardiovascular disease risk factors are less pronounced in younger samples than in comparable older samples (43, 44). Given the high variability in LTL between individuals from birth onwards (45), it is unlikely that variation in LTL was too limited by age 31 years in our sample for detecting an association with 25(OH)D concentrations. Rather, if the previous results were not chance findings (24, 25), it may be that a 25(OH)D-LTL association emerges later in the life course than young adulthood, and our findings do not preclude the possibility (or confirm) that such an association would be observable in our study sample at older ages.

We have extended a previous analysis of the cross-sectional associations of adiposity measures with LTL within this cohort by examining potential mediation of the BMI-LTL association by 25(OH)D and CRP (26). The association of BMI with LTL in these data is consistent with some, but not all, studies that have examined this issue to date (46). The disparate findings may have arisen from the wide diversity of characteristics within and between different study samples used (26). The fact that the association of BMI with LTL was attenuated somewhat but remained following adjustment for CRP suggests that inflammation could, at least in part, mediate any effects that higher adiposity may have on increased telomere degradation, if this association represents a causal relationship (we were unable to infer causality between adiposity and LTL in this study). However, CRP measurement provides information on only 1 aspect of a multitude of inflammatory pathways that may link LTL with adiposity. It would therefore be of interest to examine the impact of adjustment for a wider set of inflammatory markers and adipokines on BMI-LTL associations observed in future epidemiologic studies.

### Strengths and limitations

This study used the largest sample to date for examining associations of 25(OH)D with LTL. To our knowledge, it is also the first to have used a nonselected population sample of both men and women.

The main limitation of this study was its cross-sectional design. At present, we lack data that would allow for longitudinal analyses with LTL measured later in the life course. With only a single 25(OH)D measurement for participants, this may not have accurately captured long-term variation in vitamin D exposure, which could have introduced regression dilution bias (i.e., any true associations with outcomes would have been biased toward the null). However, 25(OH)D measures have been shown to correlate over several years (47), and our use of a single measurement is consistent with previous studies reporting associations of 25(OH)D with LTL (24, 25).

### Conclusions

Despite the lack of association of 25(OH)D with LTL among young adults in this study, it is possible that an association could become more pronounced with variation in 25(OH)D exposure over further decades of life, into middle and old age. This would explain disparities between this study and previous findings from samples of older women (24, 25). Given the implications of telomere length as an independent risk factor for chronic disease and the scope of the modifiability of 25(OH)D concentrations in populations (particularly those at northerly latitudes) (48), more studies of associations between 25(OH)D and LTL are warranted. Ideally, these studies would use a prospective design with measures of LTL at several time points across the life course, to assess potential associations with the rate of telomere shortening. The use of 25(OH)D-determining genetic variants in Mendelian randomization analyses could also help investigators to assess whether there is a long-term causal role for vitamin D status in the maintenance of telomere length (49, 50). Several variants suitable for this purpose have been identified for 25(OH)D and used in Mendelian randomization analyses to assess the relationship of vitamin D to other outcomes (51, 52). The increasing availability of LTL data on participants in large cohort studies could make this approach feasible in the future. Prospective and genetic epidemiology studies with more data on LTL and inflammatory markers will also help to elucidate the role(s) that adiposity and inflammation may play in the maintenance of telomere length, and the possible downstream consequences for chronic disease and mortality risk.

## Supplementary Material

Web Material
