# Supplementary material for: 25-Hydroxyvitamin D Concentration and Leukocyte Telomere Length in Young Adults: Findings From the Northern Finland Birth Cohort 1966
Source: Am J Epidemiol. 2016 Jan 23;183(3):191–8. doi: 10.1093/aje/kwv203 (PMC4724094; doi:10.1093/aje/kwv203)
Supplement: Web Material [file supp_183_3_191__index.html]

25-Hydroxyvitamin D Concentration and Leukocyte Telomere Length in Young Adults: Findings From the Northern Finland Birth Cohort 1966 — Web Material 

# 25-Hydroxyvitamin D Concentration and Leukocyte Telomere Length in Young Adults: Findings From the Northern Finland Birth Cohort 1966

## Web Material

Web Material

- Web Material - Pdf file
