# Supplementary material for: 25-Hydroxyvitamin D Concentration and Leukocyte Telomere Length in Young Adults: Findings From the Northern Finland Birth Cohort 1966
Source: Am J Epidemiol. 2016 Jan 23;183(3):191–8. doi: 10.1093/aje/kwv203 (PMC4724094; doi:10.1093/aje/kwv203)
Supplement: Web Material [file supp_kwv203_kwv203supp.pdf]

25-Hydroxyvitamin D and Leukocyte Telomere Length in Young Adults: Findings From the Northern Finland Birth Cohort 1966

Web Appendix

25(OH)D assay

Data on the 25(OH)D assay were received in four stages, the first in March 2008 and the last in August 2009. The order of sampling by batch was correlated with original date of storage of serum samples in the Finnish laboratory in 1997. During the period of study sample assessment, the laboratory took part in five evaluations as part of the Vitamin D External Quality Assessment Scheme (http://www.deqas.org/) (1).

Materials and chemicals

25(OH)D_2 (cat. no. 17937) and 25(OH) D_3 (cat. no. H4014) standards were obtained from Sigma Chemical Company. The internal standard of 26,27 hexadeuterium 25(OH)D_3 was purchased from Synthetica AS, Oslo, Norway. High-performance liquid chromatography-grade methanol, propan-2-ol, acetonitrile and tetrahydrofuran were obtained from VWR International (Radnor, PA, USA). Ultra-pure deionised water (>18.2 MΩ/cm) was obtained from a Millipore AFS 50 EDI unit. Isolute C18(EC) 200 mg 3mL reservoirs were purchased from Kinesis Ltd. A stream of nitrogen was provided by a Peak Scientific nitrogen generator, at 40°C in a Techne Dryblock DB3.

LC-MS/MS system

A Waters Acquity Ultra Performance Liquid Chromatography system was used to separate the peaks of interest. Chromatographic separation was achieved using a Waters SunFire C18 (3.5µm 100mm x 2.1mm id) analytical column fitted with a guard column. Column life was improved by fitting a 10mm C18 guard column. Column temperature was maintained at 40°C. Mass detection was based on the following parameters. A Waters Premier XE with a Z spray source, in electron spray ionization (ESI)-positive multiple reaction model, was maintained at a source temperature of 130°C. Desolvation temperature was set at 250°C, gas flow at 950L/h and argon collision gas set at 0.3mL/min. QuanLynx software was used to calculate relative retention time for peak identification and peak area ratios with internal standardisation for quantitation. Mobile phase A contained 2mmol/L ammonium acetate (supplied by VWR International) in 0.1% formic acid (Fluka Chemical Company). Mobile phase B contained 2mmol/L ammonium acetate in methanol containing 0.1% formic acid. A binary step gradient was used to clean the column of any late eluting peaks. Elution of the vitamins was achieved using 84% B at 0.4 ml/min for 3.5 mins and then switching to 100% B for a further minute before returning to 84%B. Injection interval was set to 5.5 mins, allowing 1 min for re-equilibration of the column before the next injection. Solvent divert was used to allow data acquisition to take place between 1.2 and 3.5 mins. Eluent was then introduced into a Waters Premier XE tandem mass-spectrometer fitted with an ESI source. The sample tray area was maintained at 15°C. Quantitation reaction model transitions were 401.1 > 383.2, 413.2 > 395.2, and 407.1 > 107.1 for 25(OH)D_3, 25(OH)D_2, and d6 25(OH)D_3, respectively. Coefficients of variation were less than 16% across the working range of the assay.

Seasonal adjustment of 25(OH)D measures

To more accurately rank individuals by their underlying 25(OH)D concentrations, 25(OH)D measures were adjusted for season of sampling. This accounts for seasonal variation in 25(OH)D_3 produced by changes in ultraviolet B radiation in sun exposure across seasons. It has been suggested that not accounting for seasonal variation in 25(OH)D results in associations with outcomes being biased towards the null (2). Total 25(OH)D was modelled against the date of blood sampling using linear regression with trigonometric sine and cosine functions, using the function

$$f(t) = \alpha + \sum_{h=1}^{4} \beta_h \sin(2h\pi t) + \sum_{h=1}^{4} \theta_h \cos(2h\pi t)$$

where α, β_h, and θ_h are estimated regression parameters and t is the date of blood sampling. The number of terms included was chosen to produce the best fit of the data after using Wald tests and graphical plots. Models were applied separately in two sections, for participants visiting the assessment centre before and after an approximate break in assessments conducted during a month over the Finnish spring holiday period. The residuals from these models were then used as a variable for season-adjusted 25(OH)D in all analyses.

Web Table 1. Univariable trends in NFBC1966 participant characteristics by fifths of the 25(OH)D distribution (*n* = 5,096), 1966–1997

	**Lowest Fifth <37.2 nmol/L**		**2nd Fifth 37.2 to 45.8 nmol/L**		**3rd Fifth 45.8 to 53.9 nmol/L**		**4th Fifth 54.0 to 62.6 nmol/L**		**Highest Fifth ≥62.7 nmol/L**		***P* Value**
Age (years)	30.78	(30.75, 30.81)	30.78	(30.75, 30.82)	30.80	(30.76, 30.83)	30.80	(30.76, 30.84)	30.80	(30.76, 30.83)	0.33
Sex (% female)	52.3	(49.2, 55.3)	52.3	(49.2, 55.4)	51.1	(48.1, 54.2)	53.9	(50.9, 57.0)	49.2	(46.1, 52.2)	0.40
BMI (kg/m2)	24.6	(24.3, 24.9)	24.5	(24.2, 24.8)	24.8	(24.4, 25.1)	24.3	(23.9, 24.6)	24.3	(23.9, 24.6)	0.005
Socioeconomic position (%)											<0.001
Farmer	4.4	(3.3, 5.8)	2.7	(1.9, 3.9)	2.5	(1.8, 3.7)	3.2	(2.3, 4.4)	3.4	(2.5, 4.7)	
Professional	24.6	(22.1, 27.4)	25.6	(23.0, 28.4)	22.9	(20.4, 25.6)	21.2	(18.8, 23.9)	22.6	(20.2, 25.3)	
Skilled worker	27.9	(25.1, 30.8)	30.5	(27.6, 33.5)	29.9	(27.0, 32.9)	28.3	(25.6, 31.3)	30.0	(27.1, 33.0)	
Unskilled worker	25.0	(22.4, 22.8)	21.7	(19.3, 24.4)	23.9	(21.4, 26.7)	25.7	(23.0, 28.5)	23.8	(21.2, 26.6)	
Other	13.6	(11.6, 15.8)	15.0	(12.9, 17.3)	16.5	(14.4, 18.9)	17.4	(15.2, 19.8)	16.1	(13.9, 18.5)	
Physical activity (% per fifth of MET score distribution)											<0.001
1	21.9	(19.5, 24.6)	20.9	(18.6, 23.6)	21.5	(19.1, 24.2)	18.8	(16.5, 21.3)	19.4	(17.1, 22.0)	
2	21.3	(18.9, 23.9)	18.7	(16.4, 21.2)	20.4	(18.1, 23.0)	17.4	(15.2, 19.9)	17.9	(15.7, 20.4)	
3	19.0	(16.7, 21.5)	24.5	(21.9, 27.2)	18.6	(16.3, 21.1)	19.0	(16.7, 21.5)	18.8	(16.5, 21.4)	
4	21.3	(18.9, 23.9)	17.4	(15.2, 19.9)	20.0	(17.7, 22.6)	21.8	(19.4, 24.4)	19.5	(17.2, 22.1)	
5	16.0	(13.8, 18.3)	18.0	(15.7, 20.4)	19.0	(16.7, 21.5)	22.5	(20.1, 25.2)	23.8	(21.3, 26.5)	
Diet quality (% per category)											0.02
0	5.7	(4.5, 7.3)	5.2	(4.0, 6.7)	5.6	(4.4, 7.1)	7.6	(6.1, 9.3)	6.8	(5.5, 8.5)	
1	22.1	(19.7, 24.8)	23.5	(20.9, 26.2)	24.3	(21.7, 27.1)	22.3	(19.9, 25.0)	23.9	(21.3, 26.6)	
2	30.7	(27.9, 35.3)	32.4	(29.6, 35.3)	29.5	(26.8, 32.4)	28.5	(25.8, 31.3)	30.7	(27.9, 33.6)	
3	25.9	(23.3, 28.7)	24.9	(22.2, 27.6)	24.5	(21.9, 27.2)	29.2	(26.4, 32.1)	25.4	(22.8, 28.2)	
4 or 5	11.5	(10.0, 13.6)	10.2	(8.5, 12.2)	12.1	(10.3, 14.3)	8.4	(6.9, 10.3)	9.2	(7.6, 11.1)	

Ever smoker (%)	60.0	(56.9, 63.0)	63.9	(60.9, 66.8)	64.4	(61.4, 67.3)	65.4	(62.4, 68.3)	62.9	(59.9, 65.8)	0.16
Cigarette consumption per day (% non-smokers, or % per tertile of use)											0.38
none	66.0	(63.1, 68.9)	62.6	(59.6, 65.6)	63.8	(60.8, 66.7)	61.7	(58.7, 64.6)	66.1	(63.1, 69.0)	
1st tertile	10.6	(8.9, 12.6)	13.2	(11.2, 15.5)	12.3	(10.4, 14.5)	14.2	(12.2, 16.5)	10.0	(8.3, 12.0)	
2nd tertile	11.9	(10.0, 14.0)	13.9	(11.9, 16.2)	12.4	(10.5, 14.5)	13.4	(11.4, 15.6)	13.4	(11.4, 15.6)	
3rd tertile	9.3	(7.7, 11.1)	8.0	(6.5, 9.8)	9.2	(7.6, 11.1)	8.4	(6.9, 10.2)	8.4	(6.9, 10.2)	
Alternative tobacco use (%)	1.4	(0.9, 2.2)	1.3	(0.8, 2.0)	1.9	(1.3, 2.9)	1.8	(1.2, 2.8)	2.6	(1.8, 3.8)	0.02
Alcohol consumption (g/day)	3.4	(3.0, 3.8)	3.5	(3.1, 4.0)	3.3	(2.9, 3.8)	3.3	(2.9, 3.7)	3.6	(3.2, 4.1)	0.51
Oral contraception use (% of females)	15.3	(12.5, 18.6)	20.2	(17.0, 23.9)	20.9	(17.6, 24.6)	24.7	(21.3, 28.5)	37.5	(33.4, 41.9)	<0.001
CRP (mg/L)	0.76	(0.68, 0.85)	0.71	(0.63, 0.80)	0.78	(0.68, 0.88)	0.78	(0.69, 0.89)	0.78	(0.68, 0.88)	0.12
LTL (T/S ratio)	1.19	(1.15, 1.22)	1.19	(1.16, 1.23)	1.17	(1.13, 1.21)	1.18	(1.15, 1.22)	1.21	(1.17, 1.25)	0.45

Abbreviations: BMI, body mass index; CRP, C-reactive protein; LTL, leukocyte telomere length; MET, metabolic equivalent of task; 25(OH)D, 25-hydroxyvitamin D; SEP, socioeconomic position.

Values are means, geometric means, or percentages (with 95% CI) of variables across fifths of the 25(OH)D distribution.

Web Table 2. Mean differences in LTL of NFBC1966 participants at mean age 31 years in those with 25(OH)D levels of 50–75 nmol/L (*n* = 2,261) and those with 25(OH)D levels of <50 nmol/L (*n* = 2,550), compared to those with 25(OH)D levels of >75 nmol/L (*n* = 285), 1966–1997

	**Mean Difference in LTL Comparing Those with 25(OH)D = 50–75 nmol/L to Those with 25(OH)D >75 nmol/L**			**Mean Difference in LTL Comparing Those with 25(OH)D <50 to Those with 25(OH)D >75 nmol/L**		
	**Coefficient**	**95% CI**	***P* Value**	**Coefficient**	**95% CI**	***P* Value**
Model 1 [a]	0. 1	-4.0, 4.4	0.97	0.2	-4.1, 4.7	0.92
Model 2 [b]	-0. 1	-4.0, 4.4	0.94	0.1	-4.2, 4.7	0.96
Model 3 [c]	-0. 2	-4.0, 4.4	0.91	0.0	-4.3, 4.6	0.99

Abbreviations: BMI, body mass index; CI, confidence interval; CRP, C-reactive protein; LTL, leukocyte telomere length; MET, metabolic equivalent of task; 25(OH)D, 25-hydroxyvitamin D; SEP, socioeconomic position.

Coefficients and 95% CIs represent mean percent difference in telomere length between 25(OH)D groups.

[a] Model 1 — adjusted for age, sex, and 25(OH)D batch.

[b] Model 2 — as for model 1 plus BMI, SEP, physical activity, diet, smoking, alcohol intake, and use of oral contraceptives.

[c] Model 3 — as for model 2 plus CRP.

Web References

1. Carter GD, Carter CR, Gunter E, Jones J, Jones G, Makin HLJ, et al. Measurement of vitamin D metabolites: an international perspective on methodology and clinical interpretation. *J Steroid Biochem Mol Biol*. 2004;89-90:467–471.

2. Wang Y, Jacobs EJ, McCullough ML, Rodriguez C, Thun MJ, Calle EE, et al. Comparing methods for accounting for seasonal variability in a biomarker when only a single sample is available: insights from simulations based on serum 25-hydroxyvitamin D. *Am J Epidemiol*. 2009;170(1):88–94.
